# An integrated approach to occupational health risk assessment of manufacturing nanomaterials using Pythagorean Fuzzy AHP and Fuzzy Inference System

**DOI:** 10.1038/s41598-023-48885-w

**Published:** 2024-01-02

**Authors:** Samaneh Salari, Mohsen Sadeghi-Yarandi, Farideh Golbabaei

**Affiliations:** Department of Occupational Health Engineering, School of Public Health, Tehran University of Medical Sciences, Tehran, Iran

**Keywords:** Health occupations, Risk factors, Engineering

## Abstract

Nanomaterials (NMs) have the potential to be hazardous owing to their unique physico-chemical properties. Therefore, the need for Health Risk Assessment (HRA) of NMs is expanding. In this study, a novel HRA was developed by the Pythagorean Fuzzy Health Risk Assessment (PFHRA) approach. Risk is considered to be the outcome of parameters including Occurrence Likelihood (OL), Potential Exposure (PE) and Toxic Effects (TE). In our proposed method, priority weights of sub-factors in Pythagorean Fuzzy-Analytical Hierarchical Process (PF-AHP) were determined by pairwise comparison based on expert judgment. After determining parameter scores, both RM and risk class (i.e., negligible, minor, major and critical) were reported as Fuzzy Inference System (FIS) output. Ultimately, a risk management strategy is presented for NMs manufacturing workplaces. This proposed method provides experts with more flexibility to express their opinions. The PFHRA approach was applied for two scenarios. The production scenario for SiNPs can create minor (5%) and major (95%) occupational health risks; the production scenario for ZnONPs can create minor (100%) concerns. However, the production SiNPs and ZnONPs utilizing the CB Nanotool technique had a major and minor risk class, respectively. The results of the present study confirmed the reliability and applicability of this approach.

## Introduction

According to the definition of the European Commission, "Nano matter is any natural, incidental, or manufactured substance that contains particles, in a non-connected state or as identifiable constituent particles in aggregates or agglomerates. Nanomaterials must have at least one dimension that is less than approximately 100 nm"^1,2^. Numerous studies have convincingly demonstrated that nanomaterials have unique physicochemical characteristics that lead to toxic effects. Creating these adverse effects cannot possible by larger particles^3,4^. Previous studies on ambient or natural nanoparticles have faced doubts regarding their possible toxic health effects^5–10^.

Nowadays, the use of nanomaterials in a various industrial processing, products, and healthcare applications has widely spread^11,12^. Due to the lack of a clear toxicological basis for setting nanomaterial-specific occupational exposure limits^13–15^, it is necessary to assess the risk of nanomaterial hazards^16–18^.

On the other hands, the rapid growth of nanotechnology has led to an increased production and use of nanomaterials^19^, which may pose potential risks to the health and safety of workers involved in their manufacturing^20^. Occupational health risk assessments are crucial in identifying and mitigating these occupational risks^21,22^. Control Banding (CB) methods such as CB Nanotool, Stoffenmanager Nano created in The Netherlands, and CB Tool from the French Agency for Food and Act, are employed as categories criteria, or "bands," for occupational and health risk assessment, which combined with parameters to determine desired levels of control measures^18,23^.

Nanotechnology has emerged as a promising field with numerous applications in various industries, including manufacturing. However, the use of nanomaterials in the workplace poses potential health risks to workers. Therefore, it is essential to assess and manage these risks effectively. However, traditional methods are uncertain and ambiguous due to the lack of safety materials datasheets or information from a literature review. In addition, there is variability and incomplete knowledge^24–27^. In this paper, we present an integrated approach to occupational health risk assessment using Pythagorean Fuzzy AHP and Fuzzy Inference System. This approach allows for a more flexible and nuanced assessment of risk factors and their interactions, providing a more comprehensive understanding of overall occupational health risk. Our approach highlight the importance of considering uncertainty and imprecision in risk assessments for nanomaterials, and the need for more sophisticated and adaptable methods in this field.

In our proposed method, the HRA for NMs was carried out in the following phases:

I. To hierarchically identify and determine the sub-factors that relate to the Occurrence Likelihood (OL) of NMs in the ambient, their Potential Exposure (PE), and the Toxic Effects (TE).
II. Assessing each sub-factor regarding its contribution to health risk using expert judgments and calculating priority weights by the Pythagorean fuzzy Analytical Hierarchy Process (PF-AHP) method for these factors that can overcome uncertainty and ambiguous data.
III. Merging all factors and determining RM and risk class by Fuzzy Inference System (FIS).

## Literature review

Many studies have used integrated AHP and FIS methods for risk assessment in mines^28^ and different project^29,30^. In this regard, a novel integrated approach, Pythagorean Fuzzy Proportional Risk Assessment, was perfectly developed by Ilbahar et al. that was integrated Fine Kinney method, Pythagorean Fuzzy Analytic Hierarchy Process (PFAHP) method, and a Fuzzy Inference System that is used for risk assessment in the risk assessment of occupational health and safety^16^. In addition, integrating AHP and FIS methods in various fields, was applied such as prioritizing of suppliers on sustainability factors^31^ and ranking environmental issues in offshore oil and gas operations^32^, Production planning^33^, and act. However, unlike the literature, this study uses the PFAHP method and a fuzzy inference system for nanomaterials health risk assessment. Topuz et al^34^. proposed an environmental risk assessment approach for engineered nanoparticles using integrated AHP and fuzzy inference rules, which systematically evaluate related risk factors and reduce uncertainty about data and information. In this study, the proposed approach was precise and helpful in determining the risk management strategies. Moreover, many studies have focused on the environmental risk assessment of various nanoparticles that is used using fuzzy logic^35,36^. Based on our research, none of the MCDM techniques in literature has been used to assess occupational and health risks of nanomaterials. Different from the literature, this study is the first to suggest a fuzzy method for the NMs' occupational and health risk assessment. Moreover, the present study has developed an accurate approach to assess occupational health risk in the manufacture of nanomaterials using PFAHP and FIS. PFAHP determines the weight of each sub-factor, considering its contribution to HRA. Finally, after determining the weight of the main factors of OL, TE, and PE, the risk class of the nanomaterial production process, including Negligible (N), Minor (Mi), Major (Ma), and Critical (C), is determined using a fuzzy inference system.

## Method

**Figure 1:**
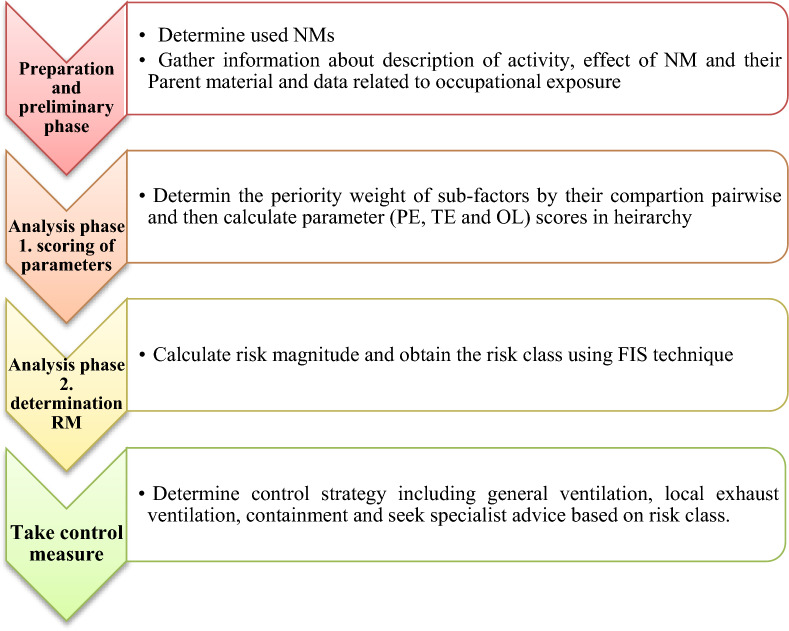
Framework of health risk assessment using PFHRA approach.

The overall framework for HRA using PFHRA is presented in Fig. 1. To obtain RM, two distinct procedures, namely the Mamdani-FIS and the PF-AHP are integrated into this proposed method. In this proposed method, factors in CB Nanotool were scored using Pythagorean fuzzy numbers, and then membership degrees of the main factors were used as input for FIS. This section, lists each technique employed in our proposed integrated method. Finally, a detailed description of the proposed integrated method is mentioned.

### CB nanotool

The conventional CB Nanotool 2.0 technique, the most popular and effective among CBs, uses probability and severity parameters directly obtained from expert opinions to establish risk classes. Zalk et al. modified this tool and presented CB Nanotool 2.0 by lowering the severity scale's maximum points^23^. The risk level is obtained using a four by four matrix shown in Table 1. The severity of the impact on human health is determined by adding scores from 13 factors (Table 2). These factors relate to the physicochemical properties and toxic effects of the parent material or the nanomaterial under study. The results of the five factors (Table 3) are summed together to get the final probability score, which considers the worker's interactions with the engineered nanomaterials under study.

**Table 1:** Risk level matrix presented by CB Nanotool 2.0^23^.

Severity	Probability			
	Extremely unlikely (0–25^a^)	Less likely (26–50)	Likely (51–75)	Probable (76–100)
Very high (76–100)	Major	Major	Critical	Critical
High (51–75)	Minor	Minor	Major	Critical
Medium (26–50)	Negligible	Negligible	Minor	Major
Low (0–25^a^)	Negligible	Negligible	Negligible	Minor

^a^Final score of severity or probability was obtained by sum of all their factors.

**Table 2:** Thirteen factors and their scores for severity parameter in CB Nanotool (retrieved from^23^).

Surface chemistry of NM, reactivity and capacity to induce free radicals	Low: 0	Medium: 5	Unknown: 7.5	High: 10	
Particle shape of NM	Spherical/compact: 0	Anisotropic: 5	Tubular/fibrous: 10	Unknown: 7.5	
Particle diameter of NM	$<$ 41–100 nm: 0	11–40 nm: 5	1–10 nm: 10	Unknown: 7.5	
Solubility of NM	Soluble: 5	Insoluble: 10	Unknown: 7.5		
Carcinogenicity of NM	Yes: 7.5	No: 0	Unknown: 5.625		
Reproductive toxicity of NM	Yes: 7.5	No: 0	Unknown: 5.625		
Mutagenicity of NM	Yes: 7.5	No: 0	Unknown: 5.625		
Dermal toxicity of NM	Yes: 7.5	No: 0	Unknown: 5.625		
Toxicity of PM	0–1 μg/m^3^: 10	2–10 μg/m^3^: 5	$<$ 41–100 μg/m^3^: 2.5	> 100 μg/m^3^:0	Unknown: 7.5
Carcinogenicity of PM	Yes: 7.5	No:0	Unknown: 3.75		
Reproductive toxicity of PM	Yes: 7.5	No:0	Unknown: 3.75		
Mutagenicity of PM	Yes: 7.5	No: 0	Unknown: 3.75		
Dermal toxicity of PM	Yes: 7.5	No: 0	Unknown: 3.75

**Table 3:** Five factors and their scores for probability parameter in CB Nanotool (retrieved from^23^).

Estimated amount of NM used during operation	$>100 \; {\text{mg}}:25$	11–100 mg: 12.5	0–10 mg: 6.25		Unknown: 18.75
Dustiness/mistiness	$>$ 15: 15	11–15: 10	6–10: 5		Unknown: 11.25
Number of employees with similar exposure	> 15: 15	11–15: 10	6–10:5		Unknown:11.25
Frequency of operation	Daily: 15	Weekly: 10	Monthly: 5	Less than monthly: 0	Unknown:11.25
Duration of operation	> 4 h: 15	1–4 h: 10	30–60 min: 5	< 30 min: 0	Unknown: 11.25

### Analytical hierarchy process

**Figure 2:**
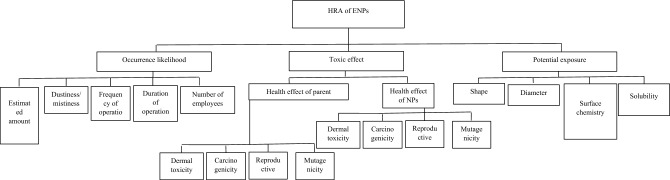
Hierarchy to evaluate sub factors of parameter in terms of their contribution to the risk of NMs.

A hierarchy of factors is presented in Fig. 2 for the HRA of NMs. AHP can consider all the factors in a hierarchical framework for their orderly arrangement. This framework clarifies their relative weights concerning health hazards. Owing to the weighting of the factors in a hierarchy, the factors demanded by experts not considered in this study can also simply be added to such an HRA approach. The main factors affecting NM health risks are addressed at the second level. Based on the comparison, the sub-factors are located at the same level in the hierarchy. This approach reduces ambiguity and uncertainty in factors by transforming expert judgments' linguistic terms into fuzzy numbers.

### Preliminaries of the Pythagorean fuzzy sets

Atanassov developed intuitionistic fuzzy sets^37^, which numerous researchers use in various fields to overcome uncertainty. In these fuzzy sets, the degree of membership and non-membership should be less than 1. Therefore^38^, has been introduced to Pythagorean fuzzy sets. Pythagorean fuzzy sets are, under some conditions, the extension of intuitionistic fuzzy sets. Pythagorean fuzzy sets are now more capable and adaptable to solving uncertainty-related issues. While the sum of membership and non-membership degrees in Pythagorean fuzzy sets can exceed 1, the sum of squares cannot, unlike intuitionistic fuzzy sets. Definition (1) expresses the following situation^16^:

#### **Definition 1**

Envision X as a fixed set. A Pythagorean fuzzy set $\widetilde{{\text{P}}}$ is an object with the properties of:

1 $$\widetilde{{\text{P}}}\cong \left\{\langle {\text{x}},{\upmu }_{{\text{P}}}\left({\text{x}}\right){\mathrm{\vartheta }}_{{\text{P}}}\rangle ;{\text{x}}\in {\text{X}}\right\}$$

where the function ${\mu }_{\widetilde{p}}\left(x\right)\to \left[\mathrm{0,1}\right]$ defines the degree of membership and ${\vartheta }_{\widetilde{p}}\left(x\right)\to \left[\mathrm{0,1}\right]$ defines the degree of non-membership of the element $x \in X to P$, respectively, and, for every $x \in X$, it holds:

$$0\le {\mu }_{\widetilde{A}}{\left(x\right)}^{2}+ {\vartheta }_{\widetilde{A}}{\left(x\right)}^{2}\le 1$$

Here, also the degree of hesitancy condition is as follows:

2 $${\pi }_{\widetilde{p}}\left(x\right)=\sqrt{1-{\mu }_{\widetilde{p}}{\left(x\right)}^{2}-{\vartheta }_{\widetilde{p}}{\left(x\right)}^{2}}.$$

#### **Definition 2**

Let $\widetilde{{\text{A}}}=\langle {\upmu }_{1},{\mathrm{\vartheta }}_{1}\rangle ,\widetilde{{\text{B}}}=\langle {\upmu }_{1},{\mathrm{\vartheta }}_{1}\rangle$ be two PFNs, and λ > 0, then the operations on these two PFNs are defined as follows:

3 $$\widetilde{{\text{A}}}\oplus \widetilde{{\text{B}}}=\left(\sqrt{{\upmu }_{1}+{\upmu }_{2}-{{\upmu }_{1}\upmu }_{2}}, {\mathrm{\vartheta }}_{1}{\mathrm{\vartheta }}_{2}\right)$$

4 $$\widetilde{{\text{A}}}\otimes \widetilde{{\text{B}}}=\left(\sqrt{{\upmu }_{1}+{\upmu }_{2}-{{\upmu }_{1}\upmu }_{2}},{\mathrm{\vartheta }}_{1}{\mathrm{\vartheta }}_{2}\right)$$

5 $$\uplambda \widetilde{{\text{A}}}=\left(\sqrt{1-{\left(1-{\upmu }^{2}\right)}^{\uplambda },{\mathrm{\vartheta }}^{\uplambda }}\right)$$

6 $${\widetilde{{\text{A}}}}^{\uplambda }=\left({\upmu }^{\uplambda },\sqrt{1-{\left(1-{\mathrm{\vartheta }}^{2}\right)}^{\uplambda }}\right)$$

#### **Definition 3**

Let $\widetilde{{\text{A}}}=\langle {\upmu }_{1},{\mathrm{\vartheta }}_{1}\rangle ,\mathrm{ i}=\left(\mathrm{1,2},\dots ,{\text{n}}\right)$ be a collection of PFNs and ${\text{w}}={\left({{\text{w}}}_{1},{{\text{w}}}_{2},\dots ,{{\text{w}}}_{{\text{n}}} \right)}^{{\text{T}}}$ be the weight vector ${\text{w}}={\left({{\text{w}}}_{1},{{\text{w}}}_{2},\dots ,{{\text{w}}}_{{\text{n}}} \right)}^{{\text{T}}}$ of ${\widetilde{{\text{A}}}}_{{\text{i}}},{\text{i}}=\left(\mathrm{1,2},\dots ,{\text{n}}\right)$ with $\sum {w}_{i} = 1$, then the Pythagorean fuzzy weighted power geometric (PFWPG) operator is:

7 $$\mathbf{P}\mathbf{F}\mathbf{W}\mathbf{P}\mathbf{G}\left({\widetilde{A}}_{1},{\widetilde{A}}_{2},\dots , {\widetilde{A}}_{n}\right)=\left({\left(1-\prod_{i=1}^{n}{\left(1-{\mu }_{i}^{2}\right)}^{{\omega }_{i}}\right)}^{1/2}-{\left(1-\prod_{i=1}^{n}{\left(1-{\vartheta }_{i}^{2}\right)}^{{\omega }_{i}}\right)}^{1/2}\right)$$

### Pythagorean Fuzzy Analytical Hierarchy Process (PFAHP)

The PFAHP steps are explained in this subsection.

**Step 1.**: The pairwise comparison matrix $R={\left({r}_{ik}\right)}_{m\times m}$ is constructed using the linguistic terms specified by experts and presented in Table 4^16^.

**Table 4:** Linguistic terms and weighting scale for PF-AHP method (retrieved from^16^).

#	Linguistic terms	IVPF numbers			
		${\mu }_{L}$	${\mu }_{u}$	${\vartheta }_{L}$	${\vartheta }_{u}$
1	Certainly low importance (CLI)	0	0	0.9	1
2	Very low importance (VLI)	0.1	0.2	0.8	0.9
3	Low importance (LI)	0.2	0.35	0.65	0.8
4	Below average importance (BAI)	0.35	0.45	0.55	0.65
5	Average importance (AI)	0.45	0.55	0.45	0.55
6	Above average importance (AAI)	0.55	0.65	0.35	0.45
7	High importance (HI)	0.65	0.8	0.2	0.32
8	Very high importance (VHI)	0.8	0.9	0.1	0.2
9	Certainly high importance—CHI	0.9	1	0	0
10	Exactly equal (EE)	0.1965	0.1965	0.1965	0.1965

*IVPF* interval-valued pythagorean fuzzy.
**Step 2:** Using Eqs. (8) and (9) to calculate the differences matrix ${\text{D}}={\left({{\text{d}}}_{{\text{ik}}}\right)}_{{\text{m}}\times {\text{m}}}$ using the lower and upper values of the membership and non-membership functions:
  8 $${{\text{d}}}_{{{\text{ik}}}_{{\text{U}}}}={\upmu }_{{{\text{ik}}}_{{\text{U}}}}^{2}-{\mathrm{\vartheta }}_{{{\text{ik}}}_{{\text{L}}}}^{2}$$
  9 $${{\text{d}}}_{{{\text{ik}}}_{{\text{L}}}}={\upmu }_{{{\text{ik}}}_{{\text{L}}}}^{2}-{\mathrm{\vartheta }}_{{{\text{ik}}}_{{\text{U}}}}^{2}$$
**Step 3:** Using Eqs. (10) and (11) to determine the interval multiplicative matrix $={\left({{\text{s}}}_{{\text{ik}}}\right)}_{{\text{m}}\times {\text{m}}}$ .
  10 $${{{\text{S}}}_{{\text{ik}}}}_{{\text{l}}}=\sqrt{{1000}^{{{\text{d}}}_{{\text{l}}}}}$$
  11 $${{{\text{S}}}_{{\text{ik}}}}_{{\text{u}}}=\sqrt{{1000}^{{{\text{d}}}_{{\text{u}}}}}$$
**Step 4:** Using Eq. (12) to calculate the determinacy value $\uptau ={\left({\uptau }_{{\text{ik}}}\right)}_{{\text{m}}\times {\text{m}}}$ of the ${{\text{r}}}_{{\text{ik}}}$.
  12 $${\uptau }_{{\text{ik}}}=1-\left({{{\upmu }_{{\text{ik}}}}_{{\text{u}}}}^{2}-{{{\upmu }_{{\text{ik}}}}_{{\text{L}}}}^{2}\right)-\left({{{\mathcal{V}}_{{\text{ik}}}}_{{\text{u}}}}^{2}-{{{\mathcal{V}}_{{\text{ik}}}}_{{\text{L}}}}^{2}\right)$$
**Step 5:** To obtain the weights matrix $T={\left({\tau }_{ik}\right)}_{m\times m}$, before normalizing using Eq. (13), multiply the determinacy degrees with the $S={\left({s}_{ik}\right)}_{m\times m}$ matrix.
  13 $${t}_{ik}=\left(\frac{{{S}_{ik}}_{u}+ {{S}_{ik}}_{l}}{2}\right){\tau }_{ik}$$
**Step 6:** Using Eq. (14) to determine the priority weight ${\upomega }_{i}$.
  14 $${\upomega }_{i}=\frac{{\sum }_{k=1}^{m}{t}_{ik}}{{\sum }_{i=1}^{m}{\sum }_{k=1}^{m}{t}_{ik}}$$

### Fuzzy Inference System (FIS)

**Figure 3:**
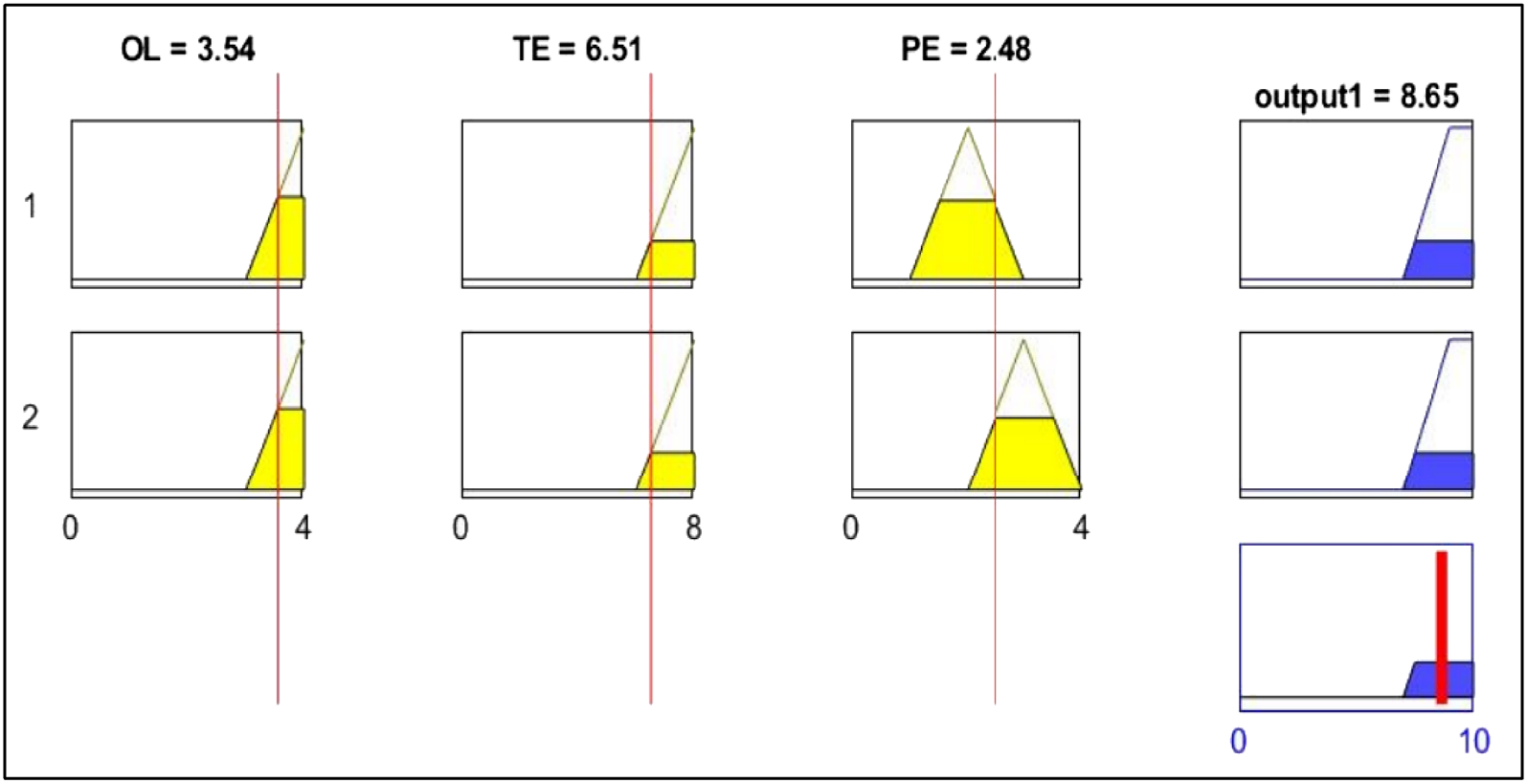
Schematic of three main factors as crisp inputs, two outputs and two fuzzy rules in FIS by MATLAB software. *OL* occurrence likelihood, *PE* potential exposure, *TE* toxic effects.

Instead of putting several risk variables into one equation and depending on multiple assumptions to determine RM with "if…then…" rules, the fuzzy inference system allows expert judgment to be combined with causal factors. The Mamdani and Sugeno types of FIS have been used in various technical and scientific applications. One of the first and most well-known algorithms in the literature is the Mamdani fuzzy model. Mamdani FIS model as shown in Fig. 3 employed in this proposed method. The FIS consists of four phases: fuzzification, knowledge base, fuzzy inference system, and defuzzification.

### Fuzzification

Fuzzification, the initial phase in the FIS process, converts crisp values into membership functions for linguistic terms of fuzzy sets. In other words, linguistic terms including very high (VH), high (H), medium (M), low (L), and very low (VL), are used to translate crisp input numbers. Membership degrees are entered into fuzzy If–Then rules.

### Knowledge base

The knowledge base is comprised of a database and rule base. The database defines the membership functions of the fuzzy sets used to generate fuzzy rules and the fuzzy if–then rules create the rule base. Fuzzy "if–then" rules, called fuzzy conditional functions, define the relationships between input and output. The following are the general if–then rule structures for the Mamdani FIS:

$$\text{If}\;\;{x}_{1} \; is \;{A}_{i1 } \; and \; {x}_{2} \; is \;{A}_{i2 } \; and \; \ldots \;and \;{x}_{n} \; is \;{A}_{in } \; then \; z \; is \;{B}_{i } \quad ({\text{for}} \;\; i=1, 2, \ldots r).$$

Where ${A}_{in}$ and ${B}_{i}$ are linguistic terms for membership function of input variable (${x}_{i}$) and linguistic terms (${x}_{i}$) for output (z), respectively, in rth rule.

### Fuzzy inference engine

The fuzzy inference unit creates a map from fuzzy inputs to fuzzy outputs based on fuzzy logic. It uses membership functions, logical operations, and if-then rules. This phase is the main section of a fuzzy system, which conducts the modeling process. It combines the facts obtained through the fuzzification phase with the rule base created in the previous phase. The Mamdani fuzzy model can be created using various fuzzy composition techniques. The most popular strategy, maximal composition, is applied in this essay. This method is described mathematically by Eq. (15).

15 $${{\mu }_{C}}_{r}\left(Z\right)=max\left[min\left[{{\mu }_{A}}_{r}\left(inpute\left(x\right)\right), {{\mu }_{B}}_{r}\left(input\left(y\right)\right),\dots \right]\right] r=\mathrm{1,2},\dots $$

where ${{\upmu }_{{\text{C}}}}_{{\text{r}}}\left({\text{Z}}\right)$ is membership of output (Z) for rth rule, ${{\upmu }_{{\text{A}}}}_{{\text{r}}}$ $, {{\upmu }_{{\text{B}}}}_{{\text{r}}}$ are membership functions input “x” and “y”, respectively.

### Defuzzification

Finally, the defuzzification process converts fuzzy sets into crisp values in Mamdani-FIS. Centroid of area (COA) is one of the most widely used defuzzification process. In Mamdani- FIS uses the defuzzification to convert fuzzy sets into crisp values. The COA technique benefit from all active rules participating in defuzzification process. Using Eq. (16), fuzzy sets in the COA approach are transformed into crisp values.

16 $${Z}_{COA}^{*}=\frac{\int {z\mu }_{A}(z)dz}{\int {\mu }_{A}(z)dz}$$

### Step of proposed integrated method

**Step 1.**: Before the analytical phase, an expert group of occupational health engineers, workers, operators, and research engineers for NMs and ENP-based products should be established. In the first, this group should be gathering information regarding the nanoparticle effects of and their parent materials, the production of NPs products, NM characteristics, and potential release paths to the ambient workplace. Next, a worksheet that resembles Table 7 needs being prepared. The expert group, as referred to in step 2, fills the pairwise comparison matrix with linguistic terms by consensus. Steps 3 to 11 of the proposed approach must be carried out by a specialist in PFAHP and FIS techniques to estimate risk magnitude based on pairwise comparison by expert group.
**Step 2.**: Form a pairwise comparison matrix and compare factors pairwise. Each factor is compared with others at in the same level based on their relative contribution to parameters.
**Step 3.**: Convert Linguistic terms into IVPF by employing the scale shown in Table 4. Then, using PF-AHP, whose processes are thoroughly discussed in "Pythagorean Fuzzy Analytical Hierarchy Process (PFAHP)" section, computes the priority weight of each factor (${\upomega }_{{\text{i}}}$).
**Step 4.**: Determine priority weight of sub-factors in the hierarchy (${\mathrm{\omega ^{\prime}}}_{{\text{i}}}$). While ${\upomega }_{{\text{i}}}$ is the weight of sub-factors in its own level, ${\mathrm{\omega ^{\prime}}}_{{\text{i}}}$ which is given in Eq. (17), displays the weight of sub-factors in the hierarchy. ${\upomega }_{{\text{i}}}$ section indicates the priority weight of i. section that is above factors in the case of being t level above it^39^.
  17 $${\mathrm{\omega^ {\prime}}}_{{\text{i}}}={\upomega }_{{\text{i}}} \times \prod_{{\text{i}}}^{{\text{t}}}{\upomega }_{{\text{i}}} \; \mathrm{ section}$$
**Step 5.**: Obtain normalized priority weight of sub-factors. Obtain normalized sub-factors weight by dividing ${\mathrm{\omega ^{\prime}}}_{{\text{i}}}$ with their maximum in the same level.
**Step 6.**: Calculate OL, PE and TE score. It is obtained from sum of all their normalized weight of sub-factors by Eq. (18). n indicates the number of their sub-factors in the hierarchy.
  18 $$parameter \; score= \sum_{i}^{n}{{\omega }^{\prime}}_{i} \; criterions \; in \; its \; sub \; level \quad i=\mathrm{1,2},\dots ,n$$
**Step 7.**: Convert normalized parameter scores to membership degrees (MD). To use OL, PE and TE scores as inputs for the FIS, they should be transformed to Trapezoidal fuzzy sets using Fig. 5 and Fig. 6.
**Step 8.**: In order to achieve RM, take the minimum of membership degree of OL, PE and TE of a NPs production process to obtain ${X}_{ijk}$ values as in following Eq. (19).
  19 $${X}_{ijk}=min\left({\mu }_{OL},{\mu }_{TE},{\mu }_{PE}\right)$$
  where i, j, and k represent OL, PE and TE, respectively; ${\mu }_{OL},{\mu }_{TE},{\mu }_{PE}$ represent MD of OL, TE and PE of NPs, respectively.
**Step 9.**: Find the values of N, Mi, Ma, and C. As part of the defuzzification technique, take the maximum of the ${X}_{ijk}$ values using Eqs. (20)–(22) that are members of the same class is shown in Table 5.

**Table 5:**
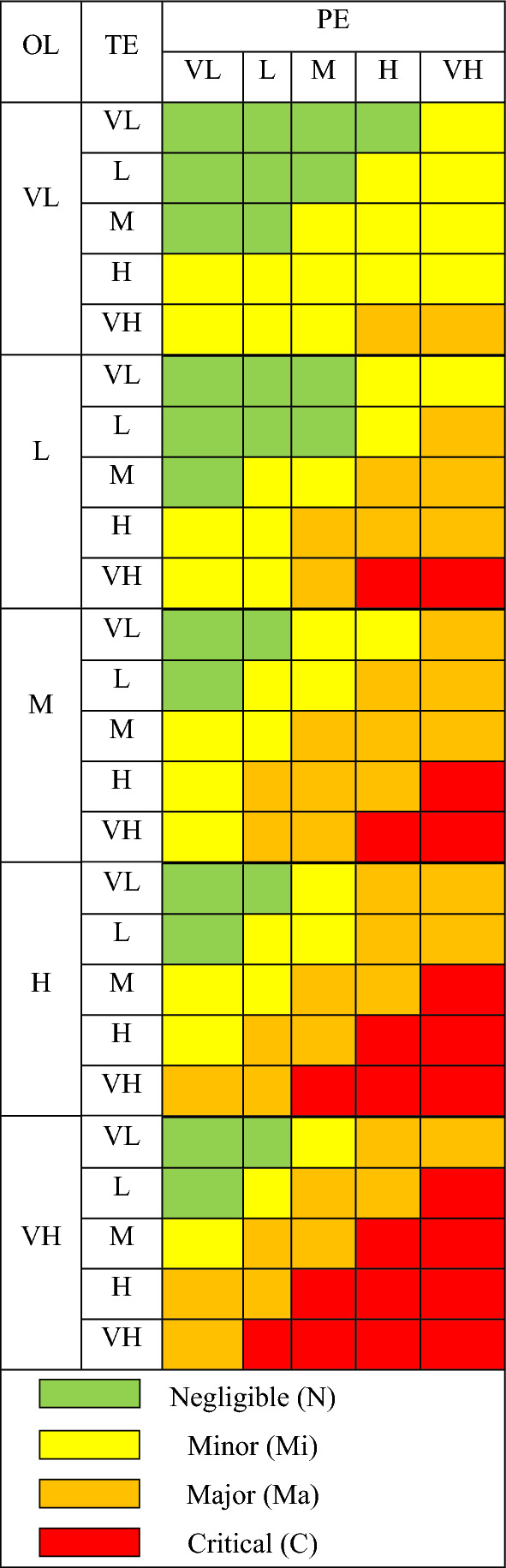
Rules of fuzzy inference system.

*OL* occurrence likelihood, *PE* potential exposure, *TE* toxic effects, *VH* very high, *H* high, *M* medium, *L* low, *VL* very low.

  20 $$N={\max}\left({x}_{ijk}\right) \forall {x}_{ijk}\epsilon N$$
  21 $$Mi={\max}\left({x}_{ijk}\right) \forall {x}_{ijk}\epsilon Mi$$
  22 $$Ma={\max}\left({x}_{ijk}\right) \forall {x}_{ijk}\epsilon Ma$$
  $$C={\text{max}}\left({x}_{ijk}\right) \forall {x}_{ijk}\epsilon C$$
**Step 10.**: Determine RM. Defuzzify the N, Mi, Ma, and C values using Eq. (23) to obtain RM.
  23 $$RM=\frac{1\times N+4\times Mi+7\times Ma+10\times C}{N+Mi+Ma+C}$$
**Step 11.**: Find the corresponding membership degrees (MD) of the NMs production process. Figure 6 is used to find the corresponding membership degrees of N, Mi, Ma, and C by RM. Finally, based on the risk class of the NMs production process, control approaches, including General ventilation, Fume hoods or local exhaust ventilation, containment and seek specialist advice is suggested for N, Mi, Ma and C class risk, respectively.

## Results and discussions

**Figure 4:**
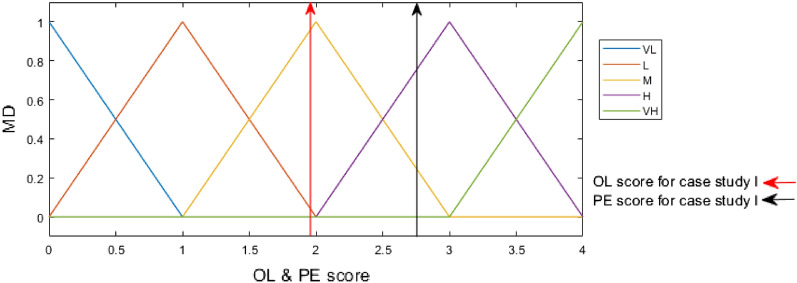
Membership functions of OL and PE inputs with OL and PE score indicators for case study I. *OL* occurrence likelihood, *PE* potential exposure, *VH* very high, *H* high, *M* medium, *L* low, *VL* very low.

**Figure 5:**
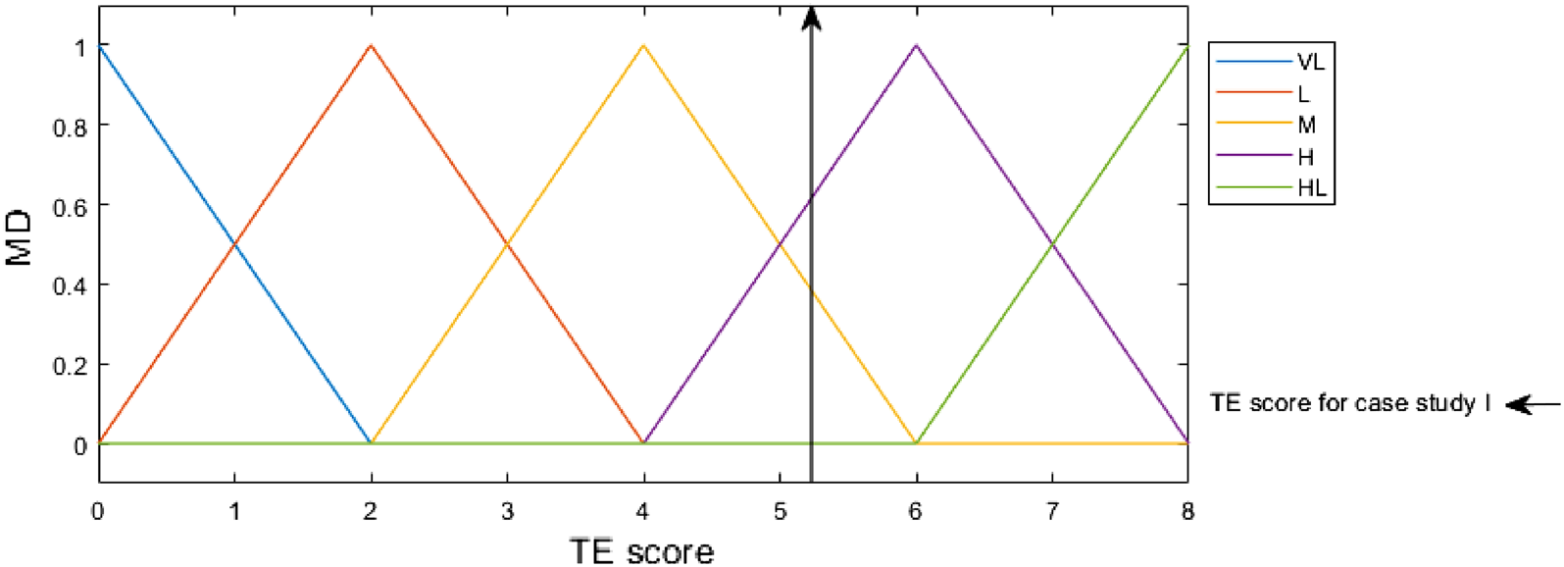
Membership functions of TE input with TE score indicator for case study I. *TE* toxic effects, *VH* very high, *H* high, *M* medium, *L* low, *VL* very low.

**Figure 6:**
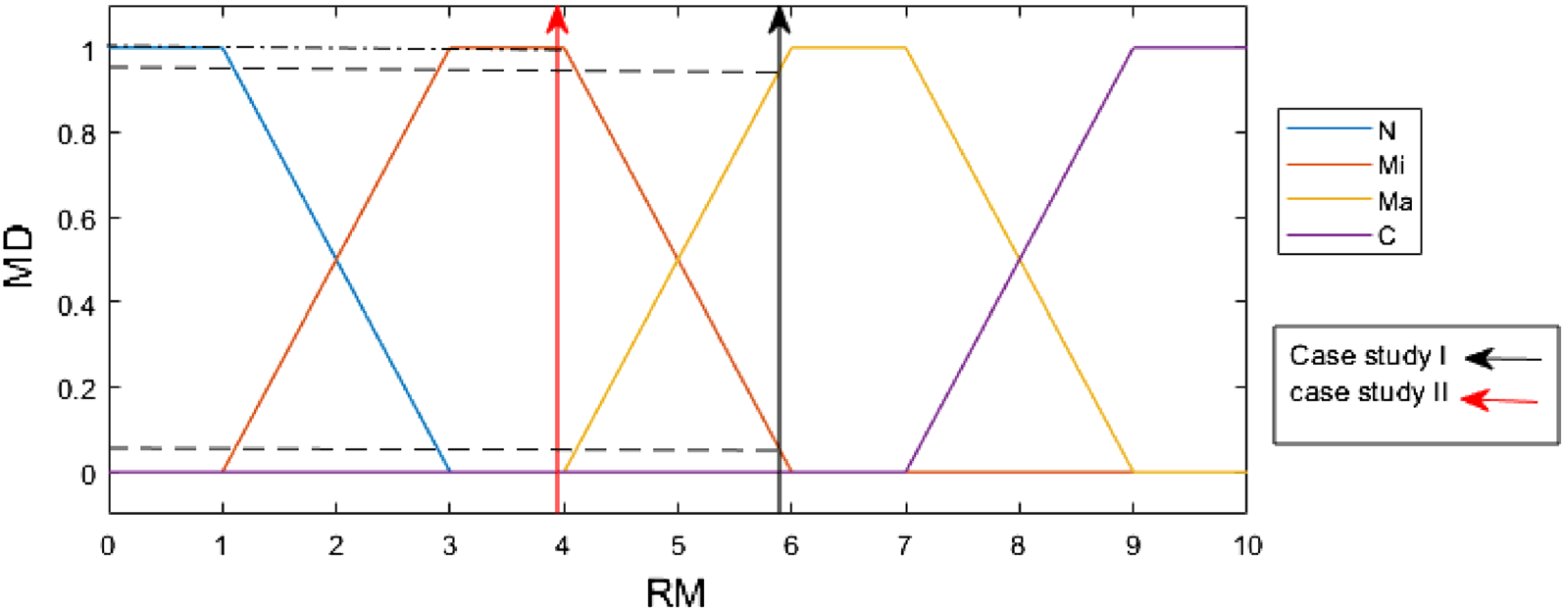
Membership functions of output with class risk indicators for case studies I and II. *MD* membership degree, *RM* risk magnitude, *N* negligible, *Mi* minor, *Ma* major, *C* critical.

The PFHRA method is applied to two case studies with two different NPs. Table 6 summarizes the overall results. CB Nanotool technique has already been introduced in “CB nanotool section”, which is then applied to case studies I and II. Their results are compared with the PFHRA method after the proposed method has been applied to assess health risks in case studies. Since information on NM production and data on OL, EP and TE parameters is still fairly limited for health risk, case studies with accessible data from the literature has been served to validate the proposed HRA method. The same assumptions as case Study I fuzzy conditional functions, define the relationships between apply to case Study II, the except that ZnONPs are produced. This manuscript does not present case Study II data due to space constraints. The scenario for case Study I involved making SiNPs into solid powder suspensions summarized in Table 7. First, the main factors at the first level are compared in pairs. Experts compare the sub-factors at the same level in pairs according to their contribution to health risk. The consistency ratios (CR) of the pairwise comparison matrices were less than 0.1, indicating the reliability of pairwise comparison by expert judgments based on the corresponding numerical values in the Classic AHP technique for the linguistic scale^40^. Table a.1–a.6 in Appendix A provide pairwise comparisons and the weights (ω) for the factors from a case study I (i.e., the main factor and sub-factors) besides their CR. Using the scale shown in Table 4, the linguistic values in these grids are transformed into IVPF numbers. PFAHP is applied to OL, TE and PE scores. The priority weight of sub-factors in the hierarchy is calculated by multiplying the weight by the weight of each factor above it. After finding the OL score, the sub-factor weights belonging to the OL factor are added together. TE and PE scores are calculated the same. OL, TE and PE scores for case study I are demonstrated in Figs. 4 and 5. Moreover, Figs. 4 and 5 are applied to determine OL, PE and TE membership degrees. After calculating N, Mi, Ma, and C values, defuzzification uses Eq. (22) to determine RM. As a result of FIS, the contribution of risk class and RM of the case studies is shown in Fig. 6.

**Table 6:** Results of using proposed method in the case studies.

NMs	RM	Negligible	Minor	Major	Critical
Case study I	5.89	0	5%	95%	0
Case study II	3.96	0	100%	0	0

**Table 7:** Summary of the case study I assumptions.

Description of activity	Scenario description: Preparing suspensions of solid nanomaterial powder for drug delivery Used nanomaterial: Mesoporous silica nanoparticles Activity classification: For handling nanoparticles in powder form
Effect of parent material	Synthesis of SiNPs using SBA-15 silica material Toxicity of PM: NIOSH REL: 0.05 mg/m^3^ TWA: 0.025 mg/m^3^/current OSHA PEL: 0.05 mg/m^3^ Carcinogen: Yes. NIOSH considers crystalline silica to be a potential occupational carcinogen Reproductive hazard: Yes Mutagen: – Dermal hazard: Yes Note: There is conflicting information, especially about mutagen and reproductive effect
Effect of NPs	*Numerous studies have shown that silica nanoparticles can damage the lungs, the nervous system, the kidneys, the liver, the liver, the heart, the cytotoxicity, and the genome^48,49^ *About 80% of research on the SiNPs toxicity revealed the toxicity of SiNPs produced using the wet approach, while only about 20% documented the toxicity of other types (such as pyrogenic and mesoporous)^48^ Carcinogen: Yes by the IARC^50^ Reproductive hazard: Yes Mutagen: Yes Dermal hazard: Yes Note: Regarding, there is conflicting information especially mutagen and reproductive effect
NPs characteristics	Surface reactivity: High Shape of NPs: Nanospheres and nanorods Diameter of NPs: 500 nm using scanning electron microscopy (SEM) Solubility: Low solubility but not insignificant water solubility
Occupational exposure information	Estimated minimum amount of substance (production capacity): Less than 10 mg/day Dustiness: 30 mg/day Number of employees with similar exposure: 1–5 persons Frequency of operation: 5 less than monthly Operation duration (per shift): 1–4 h

The SiNPs production was estimated to cause minor (5%) and major (95%) occupational health concerns. In regard to ZnONPs production, it was minor (100%) (Table 6 and Fig. 6). Therefore, Manufacturing ZnoNPs and SiNPs require local exhaust ventilation and containment devices, respectively. While, using the CB Nanotool technique, were determined major and minor for a case study I and II, respectively. Compared to the CB Nanotool, the proposed method uses fuzzy sets to scale the RM and allows the results to report membership degrees for each risk class.

The main sub-factors of the TE, PE, and OL factors based on their weight are carcinogenicity (0.41), surface chemistry and shape features (0.37), and estimated amount (0.51), respectively. Among the factors related to SiNPs, the toxic effect (score = 5.3) is highly contributive to health risk because of its high carcinogenicity. The results of the health risk assessment of our proposed method involving ZNO and SiNP nanoparticles demonstrated that SiNP poses much more serious risk to occupational health than ZnO. In this regard, previous studies have confirmed that SiNPs manufacturing is more crucial than ZnONPs^41,42^. In addition, Numerous studies have demonstrated that the production process for SiNPs carries a high level of occupational health risk^43,44^. On the other hand, the risk of exposure to ZnO is regarded as low in numerous pieces of research on risk analysis^45,46^.

These results are demonstrated that our proposed method is reliable and informative. Since the risk assessment precedes risk management applications, risk magnitude must be properly addressed to decide what to do afterward. Therefore, the results provided by the proposed approach can facilitate decision-making related to risk management strategies. This proposed integrated method answers to essential questions regarding HRA for NMs. I) What is the significance of each factor for risk; II) What are the risk class and their membership degrees; and III) What risk management strategies should be employed?

## Conclusion

Occupational health and safety require systematic analysis to protect employees from dangers that might be caused by nanomaterial exposure in the workplace. In this study, PFHRA, a combination of PF-AHP and FIS, is proposed and then PFHRA method applied for the HRA of ZnONPs and SiNPs. On the other hand, a comparison with CB Nanotool is conducted revealing that the proposed method provides reliable outcomes containing more information about uncertainty of decision makers. This should be very handy for risk managers to establish their strategies to reduce the risk.

In conclusion, the integrated approach of Pythagorean Fuzzy AHP and Fuzzy Inference System provides a more comprehensive and accurate assessment of health risks associated with the use of nanomaterials in manufacturing. The results of this study demonstrate the potential benefits of using this approach in occupational health risk assessments. However, the practical challenges and limitations of implementing this approach in real-world settings need to be addressed to ensure its successful adoption. Overall, this study highlights the importance of considering multiple factors and utilizing advanced techniques in occupational health risk assessments to protect the health and safety of workers in the manufacturing industry.

### Strengths of the study

Health Risk Assessment of NMs in the workplace is crucial because NMs might be dispersed in the work environment. In this regards, the proposed method provides a comprehensive and systematic method for assessing the occupational health risks associated with the manufacturing of nanomaterials. The use of Pythagorean Fuzzy AHP allows for the consideration of uncertainty and imprecision in the decision-making process, while the Fuzzy Inference System enables the integration of multiple risk factors and their interactions. PFHRA method can be applied to assess risk of the different NMs production in laboratories and industrial workplaces. Output of the proposed approach suggests control strategy should be used for the workplace. The control strategy is ultimately realized based on risk class by this method.

This is important for risk management of work environment. Therefore, our proposed method can be used with high reliability and correctly to assess the risk of the workplace. This proposed method is used in all workplaces where nanomaterials are produced, such as laboratories, industrial for nanomaterial production, etc.

Moreover, the results of the present study can create a novel scientific perspective in the field of health risk assessment due to occupational exposure to nanomaterials.

### Limitations of the study

Although the PFHRA method in this work provides several of the advantages listed above, there are still some limitations and a need for additional research in the following claims. However, further research is needed to validate the proposed approach using additional case studies and to refine the methodology based on feedback from industry experts. On the other hands, three parameters, OL, PE, and TE, were considered in this method to determine the RM. However, there are additional sub-factors for these parameters that are likely to influence the RM for NMs. In this regard, the study of Nina Elizabeth Landvik and et al. might be used^47^. In this manuscript, criteria for grouping NMs have been comprehensively introduced in order to facilitate hazard and risk assessment of NMs. They could be taken into account in the risk assessment for further study. To lessen uncertainty in HRA, more data (e.g., NMs data) must be collected, and another approach must be developed (e.g., incorporating dynamic techniques machine learning for example fuzzy neural network model). The expansion of the proposed approach with other fuzzy set types, such as neutrosophic sets, Fermatean fuzzy sets, circular intuitionistic fuzzy sets and Decomposed fuzzy sets. These viewpoints may be employed in future work for a more comprehensive quantitative and qualitative HRA for NMs.

While the Pythagorean Fuzzy AHP and Fuzzy Inference System offer a more comprehensive and accurate assessment of health risks associated with the use of nanomaterials in manufacturing, there may be practical challenges in implementing these methods in a workplace environment. For example, there may be limitations in data availability or accessibility, or challenges in communicating complex risk assessment results to decision-makers in the industry. Addressing these challenges and developing strategies to overcome them will be critical to ensuring the successful implementation and adoption of this integrated approach in real-world settings.

### Supplementary Information

Supplementary Information.
