# Supplementary material for: An integrated approach to occupational health risk assessment of manufacturing nanomaterials using Pythagorean Fuzzy AHP and Fuzzy Inference System
Source: Sci Rep. 2024 Jan 2;14:180. doi: 10.1038/s41598-023-48885-w (PMC10762155; doi:10.1038/s41598-023-48885-w)
Supplement: Supplementary file 1 — Supplementary Information. [file 41598_2023_48885_MOESM1_ESM.docx]

**Appendix A:**

In this Appendix, Table a.1 to a.6 provides pairwise comparisons and the weights (ω) for the factors from a case study I (i.e., the main factor and sub-factors) besides their CR.

The abbreviations in the table a.1 to a.6 are as follows: CLI: Certainly Low Importance, VLI: Very Low Importance, LI: Low Importance, BAI: Below Average Importance, AI: Average Importance, AAI: Above Average Importance, HI: High Importance, VHI: Very High Importance, CHI: Certainly High Importance and EE: Exactly Equal.

Table a. 1 Pairwise comparison of sub-factor of OL for Case Study I

|  | Estimated amount | Dustiness | Frequency | Duration | Number of employees | $\boldsymbol{\omega}$ |
| --- | --- | --- | --- | --- | --- | --- |
| Estimated amount | AAI | EE | VHI | VHI | HI | 0.51 |
| Dustiness | EE | BAI | LI | LI | HI | 0.11 |
| Frequency | HI | VLI | EE | LI | AI | 0.12 |
| Duration | HI | VLI | HI | EE | AAI | 0.21 |
| Number of employees | LI | LI | AI | BAL | EE | 0.05 |
| CR= 0.057 | | | | | | |

Table a. 2 Pairwise comparison of sub-factor of Health effect of NPs for Case Study I

|  | Dermal toxicity | Carcinogenicity | Reproductive | Mutagenicity | $\boldsymbol{\omega}$ |
| --- | --- | --- | --- | --- | --- |
| Dermal toxicity | EE | LI | LI | VLI | 0.072 |
| Carcinogenicity | HI | EE | HI | AI | 0.41 |
| Reproductive | HI | LI | EE | LI | 0.23 |
| Mutagenicity | VHI | AI | HI | EE | 0.24 |
| CR = 0.079 | | | | | |

Table a. 3 Pairwise comparison of sub-factor of Health effect of parent material for Case Study 1

|  | Dermal toxicity | Carcinogenicity | Reproductive | Mutagenicity | $\boldsymbol{\omega}$ |
| --- | --- | --- | --- | --- | --- |
| Dermal toxicity | EE | LI | LI | LI | 0.04 |
| Carcinogenicity | HI | AI | AI | EE | 0.49 |
| Reproductive | HI | AI | EE | AI | 0.16 |
| Mutagenicity | HI | EE | AI | AI | 0.30 |
| CR= 0.003 | | | | | |

Table a. 4 Pairwise comparison of sub-factor of PE for Case Study 1

|  | Shape | Diameter | Surface chemistry | Solubility | $\boldsymbol{\omega}$ |
| --- | --- | --- | --- | --- | --- |
| Shape | EE | HI | AI | HI | 0.37 |
| Diameter | LI | EE | LI | LI | 0.07 |
| Surface chemistry | AI | HI | EE | HI | 0.37 |
| Solubility | LI | HI | LI | EE | 0.20 |
| CR= 0.083 | | | | | |

Table a. 5 Pairwise comparison of main factors for Case Study 1

|  | Occurrence likelihood | Toxic effect | Potential exposure | $\boldsymbol{\omega}$ |
| --- | --- | --- | --- | --- |
| Occurrence likelihood | EE | VLI | VLI | 0.04 |
| Toxic effect | VHI | EE | HI | 0.55 |
| Potential exposure | VHI | LI | EE | 0.41 |
| CR= 0.094 | | | | |

Table a. 6 Pairwise comparison of factors in second level for Toxic effect for Case Study 1

|  | Health effect of parent materials | Health effect of NPs materials | $\boldsymbol{\omega}$ |
| --- | --- | --- | --- |
| Health effect of parent materials | EE | BAL | 0.35 |
| Health effect of NMs | AAL | EE | 0.65 |
| CR= 0.075 | | | |
